# Clinical language search algorithm from free-text: facilitating appropriate imaging

**DOI:** 10.1186/s12880-022-00740-6

**Published:** 2022-02-04

**Authors:** Gunvant R. Chaudhari, Yeshwant R. Chillakuru, Timothy L. Chen, Valentina Pedoia, Thienkhai H. Vu, Christopher P. Hess, Youngho Seo, Jae Ho Sohn

**Affiliations:** 1 Center for Intelligent Imaging, Radiology and Biomedical Imaging, University of California San Francisco (UCSF), 505 Parnassus Ave, San Francisco, CA 94143 USA; 2 George Washington School Medicine and Health Sciences, 2300 I St NW, Washington, DC 20052 USA; 3 Illinois School of Medicine, 1853 W Polk St, Chicago, IL 60612 USA

**Keywords:** Natural language processing, Information retrieval, Appropriateness criteria, Term frequency-inverse document frequency

## Abstract

**Background:**

The comprehensiveness and maintenance of the American College of Radiology (ACR) Appropriateness Criteria (AC) makes it a unique resource for evidence-based clinical imaging decision support, but it is underutilized by clinicians. To facilitate the use of imaging recommendations, we develop a natural language processing (NLP) search algorithm that automatically matches clinical indications that physicians write into imaging orders to appropriate AC imaging recommendations.

**Methods:**

We apply a hybrid model of semantic similarity from a sent2vec model trained on 223 million scientific sentences, combined with term frequency inverse document frequency features. AC documents are ranked based on their embeddings’ cosine distance to query. For model testing, we compiled a dataset of simulated simple and complex indications for each AC document (n = 410) and another with clinical indications from randomly sampled radiology reports (n = 100). We compare our algorithm to a custom google search engine.

**Results:**

On the simulated indications, our algorithm ranked ground truth documents as top 3 for 98% of simple queries and 85% of complex queries. Similarly, on the randomly sampled radiology report dataset, the algorithm ranked 86% of indications with a single match as top 3. Vague and distracting phrases present in the free-text indications were main sources of errors. Our algorithm provides more relevant results than a custom Google search engine, especially for complex queries.

**Conclusions:**

We have developed and evaluated an NLP algorithm that matches clinical indications to appropriate AC guidelines. This approach can be integrated into imaging ordering systems for automated access to guidelines.

**Supplementary Information:**

The online version contains supplementary material available at 10.1186/s12880-022-00740-6.

## Background

Evidence-based medicine in radiology helps ensure that patients undergo appropriate examinations that maximize diagnostic benefit while minimizing cost, radiation exposure, and overdiagnosis [1]. A key resource that compiles evidence-based imaging recommendations for diagnostic evaluation is the American College of Radiology (ACR) Appropriateness Criteria (AC). At the time of writing, the AC consist of 12 broad categories with a total of 205 topic documents, each giving imaging recommendations for a unique symptom or disease [2]. The AC are reviewed and regularly updated by panels of clinicians who are considered experts in each listed clinical indication or disease [2]. Although the AC are comprehensive and carefully maintained, they are highly underutilized by clinicians and trainees. Only 21% of medical students and 2.4% of attending physicians reference the criteria when ordering radiology studies [3, 4]. Clinicians prefer faster and easier free-text based search methods like UpToDate and MD Consult [4] that are less rigorously vetted by radiologists, reducing the positive impact that the AC can have on patient care.

A major barrier to wider use of the AC is arguably the time and effort required to manually search for guidelines when ordering radiology studies. Existing AC access tools, such as ACR Select [5] and AC search [6], rely on rigid text inputs and/or clinician selection from categorical lists of symptoms. A similar tool used for ordering cardiology studies required clinicians 137 ± 360 s to use on average [7]. A more automated AC access method that requires less clinician time and effort is likely to increase AC usage [8, 9].

We propose a solution to overcome the identified disadvantage of the time and effort required for AC use, a solution that is implementable within existing clinical workflow. Ordering clinicians document most information relevant to determining appropriate imaging studies within the clinical indications section of imaging study orders in the Electronic Health Record (EHR). We hypothesize that these digital free-text clinical indications, which are an existing part of clinical workflow, can be used to automatically provide ordering clinicians with appropriate imaging recommendations from the AC using natural language processing (NLP).

Existing NLP search engines offer poor performance on raw clinical indications from radiology reports. These algorithms are designed to handle short and simple queries while clinical indications are often highly technical, poorly structured, of variable brevity, and with some irrelevant content. However, recent advancements in NLP have improved the accuracy of semantic matching, especially in biomedical contexts [10]. A new NLP algorithm called sent2vec [11] is an extension of word2vec and has shown promise in biomedical semantic matching [12]. Additionally, word2vec has been optimized for searching by incorporating Term Frequency-Inverse Document Frequency (TF-IDF) features, which encode information about a document’s relation to the corpus [13]. In this study, we show that these new NLP techniques can be amalgamated and finetuned to interpret clinical indications text.

### Motivation

Though the ACR Appropriateness Criteria (AC) are an evidence-based database for determining appropriate imaging, they are underused due to the time and effort of manually searching them. We propose an NLP approach for suggesting appropriate imaging at the time of ordering imaging. Specifically, we combine domain-specific sentence embeddings and AC corpus information to allow for searching AC topic documents from free-text clinical indications in imaging study orders.

## Methods

### Data

This was an algorithm development and evaluation study involving simulated and clinical radiology report datasets. The AC corpus (n = 205 topic documents) was extracted from the ACR website (link). Then, text for each topic document was tokenized and lemmatized using the python *nltk* library [14] to create a set of document bodies. Separately, titles and variants for each document were extracted to create a set of document headers. The simulated evaluation dataset of 410 indications (Testing Dataset 1) was created by a medical student and a PGY-5 radiology resident under the supervision of a board-certified radiologist. This testing dataset contained two indications for each AC corpus topic document, including pediatric topics. Radiology reports used for evaluation in Testing Dataset 2 were retrospectively collected following Institutional Review Board approval and consent waiver from a single tertiary academic medical institution.

### Text preprocessing

In all cases, the raw query was preprocessed by solving abbreviations, tokenizing, and removing stop words and punctuations. To solve abbreviations, the Radiopedia list of ~ 3000 abbreviations [15] was extracted, processed, and edited to discard irrelevant and ambiguous abbreviations. Expanded abbreviations were added to the query.

### Algorithm development

**Fig. 1:**
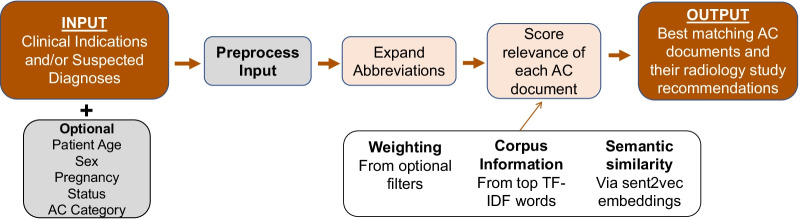
Algorithm flow diagram. This figure is a flow diagram of the algorithm’s backend from input to output. TF-IDF (Term frequency-inverse document frequency); AC (Appropriateness Criteria)

An overview of our algorithm’s backend is outlined in Fig. 1. All code is available at https://bit.ly/3giZwSa. Our algorithm’s overall complexity is O(n), with n being the number of words in the search query.

### AC document ranking score

The semantic similarity aspect of our algorithm uses sent2vec [11], an extension of word2vec [16]. We implemented sent2vec with unigrams and bigrams. Our model was trained on the open-source PubMed and MIMIC-III datasets [17], mimicking the approach of the BioSentVec model (https://bit.ly/2X7ZB1W) [18]. After training, the model was used to embed each AC document into three vectors, one for the document’s body, one for its header, and one for its top 50 TF-IDF features (see below). Each document’s ranking score, $S_{i}$, for a given query, $q$, was calculated by the following:

$$S_{i} = H_{q,i} + B_{q,i} + \beta T_{q,i}$$

where $\beta$ is the weight given to the TF-IDF score, and $H_{q,i}$, $B_{q,i}$, $T_{q,i}$ are the cosine similarities between the query’s embeddings vector and document $i$’s header, body, and TF-IDF feature vectors, respectively.

### Term frequency-inverse document frequency features

A term frequency-inverse document frequency (TF-IDF) model [19, 20] was created from raw AC documents using *TfidfVectorizer* in *scikit-learn* in python, with unigrams, bigrams, and trigrams. This model calculates a score for each word/phrase based on its frequency in a document relative to the corpus. Each document’s top 50 features were embedded into one vector using the sent2vec model.

### Testing dataset 1: simulated radiology indications dataset

To comprehensively evaluate retrieval of each AC document, we generated a query dataset of two clinical indications for each of the 205 AC documents, one simple indication and one complex indication with distractors and synonymous wording similar to those in clinical indications (examples in Additional file 1: Table S1). To quantify the quality of search result ranking from our simulated queries, we used normalized discounted cumulative gain (NDCG) [21]:

$$NDCG = \frac{{ \mathop \sum \nolimits_{i = 1}^{n} \frac{{rel_{i} }}{{log_{2} \left( {i + 1} \right)}} }}{{\mathop \sum \nolimits_{i = 1}^{n} \frac{{REL_{i} }}{{log_{2} \left( {i + 1} \right)}}}}$$

where $n$ is number of unique AC documents, $i$ is the search result rank, $rel_{i}$ is the relevance of result $i$, and $REL_{i}$ is the maximum relevance of result $i$.

Relevance was calculated by first tagging each AC document with one or more of the following tags: vascular disease, infection/inflammation, neoplasm, congenital, trauma, surgical, and many etiologies/topics (e.g. chest pain). Then, $rel_{i}$ was calculated by number of matching tags between query and search result $i$. Maximum possible relevance ($REL_{i}$) was calculated by sorting the query’s relevance for all results. An NDCG of 1 would indicate perfect search result ranking.

### Testing dataset 2: radiology report clinical indications dataset

**Fig. 2:**
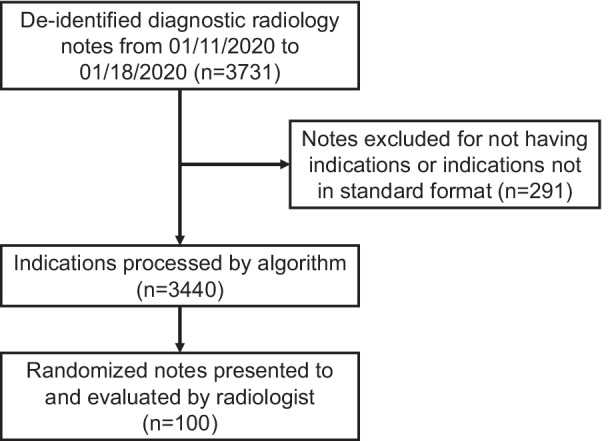
Flowchart on institutional radiology report dataset. This figure is a flow diagram of patient indication inclusion, exclusion, and processing in evaluating our algorithm on the clinical radiology report indications

To test the algorithm’s performance in clinical workflow, we extracted a dataset (n = 3731) of de-identified radiology notes from our department of radiology from 01/11/2020 to 01/18/2020 (Fig. 2). Diagnostic radiology reports from all study types except chest x-rays were extracted consecutively and comprehensively with limited exclusion criteria as specified below to minimize selection bias and simulate real clinical workflow. Chest X-ray reports were not collected as indications are frequently too simple (e.g. “fever”) or not clinically relevant. Clinical indications section text was automatically extracted from this dataset using pattern matching. Some reports (n = 291; 7.8%) were excluded because they had blank indication text or the radiology report did not follow our institution’s standard format. The resulting n = 3440 radiology report clinical indications were run through our algorithm and top 10 predictions were aggregated. A random subset of 100 indications and algorithm outputs was evaluated by a radiologist who clinically determined whether each indication had none, one, or multiple appropriate AC documents, and ranked which (if any) of the algorithm outputs were correct.

### Custom google search engine

Using Google’s Programmable Search feature, we created a custom Google search engine that was constrained to searching on the AC documents. This engine was programmed to search only on web pages with the prefix “https://acsearch.acr.org/docs/”, which corresponded to the 205 AC topic document pages. We ran a randomly chosen subset of Testing Dataset 1 (n = 100 indications) through the custom Google search and our algorithm.

### Statistical Analysis

All values reported are means unless otherwise noted. To compare performance between simple and complex simulated indications, the Mann–Whitney U test was used for NDCG values and ground truth rank values, a chi-squared test for Top 3 values, and a two sample Kolmogorov Smirnov test for the cumulative frequency curves. For the NDCG analysis, the non-parametric Kruskal–Wallis H-test was used to compare performance among AC categories. The Friedman rank test was used to compare performances between our proposed algorithm and a custom Google search. All statistical analyses were conducted in python using the *scipy* package. Statistical significance was defined as *p* < 0.05.

## Results

### Datasets and summary statistics

The training dataset for the semantic similarity model consisted of 223 million sentences extracted from PubMed and MIMIC-III databases. The sent2vec model, a continuous bag-of-words algorithm, was trained using unigrams and bigrams from this dataset.

Testing dataset 1 consisted of 410 simulated indications covered all 205 documents of the AC. On average, 32% of words in simple indications were keywords from the document’s title, while only 9% were keywords in complex indications. Additionally, all of the complex indications, which had been generated to resemble true radiology report indications, had some form of a distractor, including one or more of: age in text form (e.g. “25 year old”), less relevant medical history (e.g. “hypertension, diabetes mellitus” for a trauma patient), less relevant social history (e.g. “40 pack-year smoker”), and synonymous wording (e.g. “kidney stones” for “Urolithiasis” document). No simple indication had these distractors.

Testing dataset 2 consisted of 100 manually annotated clinical indications from radiology reports that covered 27% of AC topic documents. Characteristics of this dataset are detailed in Table 1.

**Table 1:** Patient and study characteristics of annotated institutional radiology report dataset

Characteristic	Proportion of dataset
**Body part scanned**	
Abdomen/pelvis	0.32
Chest/breast	0.15
Extremity	0.08
Head	0.30
Spine	0.13
Full body	0.02
**Age** (mean: 49.9 ± 22.1 years)	
Under 13	0.07
13–65	0.63
Over 65	0.30
**Gender**	
Male	0.44
Female	0.56
**Scan type**	
CT	0.43
MRI	0.47
US	0.08
NM	0.02

Total size of the dataset was 100 indications. For age, mean ± one standard deviation is also reported. Ultrasound (US), Nuclear medicine (NM)

### Algorithm evaluation results

**Fig. 3:**
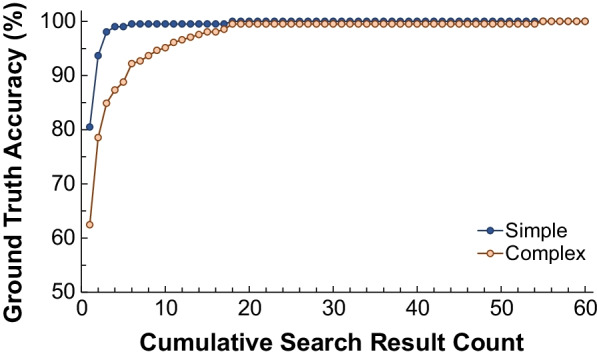
Accuracy on simulated indications dataset. This figure is a cumulative graph of the percentage of indications with ground truths ranked as the top search result to those within the top 60 search results. It shows a significant difference between algorithm performances on simple and complex simulated indications (*p* < 1e−10, two sample Kolmogorov Smirnov test)

**Fig. 4:**
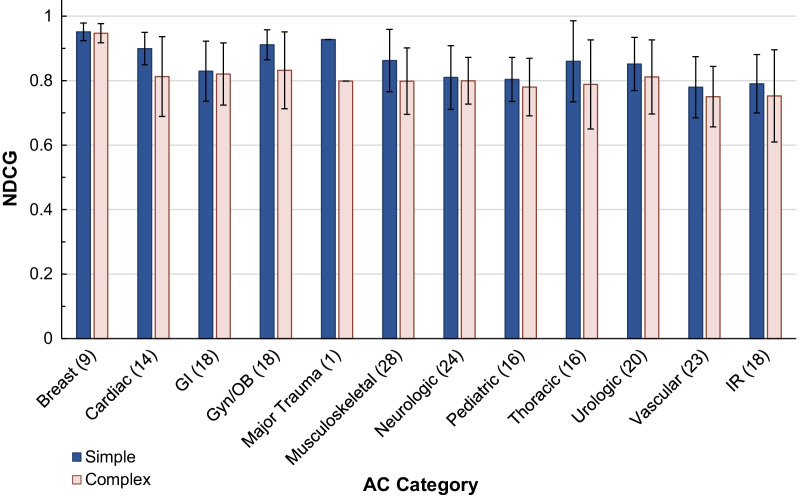
Search ranking relevance on generated indications dataset. This figure is a bar graph of the average NDCG on simple and complex queries in all 12 categories. Error bars indicate 1 standard deviation. The number of documents (and therefore queries) for each category is in parentheses. There is a significant difference between the NDCG values among categories (*p* = 0.00054, Kruskal–Wallis H-test). Note: ‘Major Trauma’ category was excluded from statistical analysis due to sample size of 1. NDCG (normalized discounted cumulative gain)

Algorithm performance was first evaluated on simulated indications in Testing Dataset 1. For simple simulated indications, the algorithm ranked the ground truth document as within the top 3 results for 98.5% of queries (Fig. 3), with an average ground truth rank of 1.36 ± 1.34 and average NDCG of 0.84 ± 0.10 (Table 2). Similarly, for complex indications, the algorithm ranked ground truth as within the top 3 for 85% of queries, with an average rank of 2.72 ± 4.79 and average NDCG of 0.80 ± 0.11. Notably, all simple queries and all but one complex query had ground truths ranked within the top 17 search results. NDCG values of complex indications were also significantly different from those of simple indications (Table 2), supporting our hypothesis that EHR-style clinical indications are more difficult to query than simple searches. Nevertheless, the high NDCG values of above 0.8 for both types of indications show that our algorithm was producing relevant search results, even for EHR-style queries. The NDCG values were significantly different across AC categories (Fig. 4, *p* = 0.013). This finding implies that some AC categories may produce better search results than others, so further category-specific finetuning may improve algorithm performance.

**Table 2:** Simulated indications dataset results

Analysis metric	Simple indications	Complex indications	*P* value
Proportion of ground truth documents in top 3	0.985	0.849	*P* < 0.0001
Average ground truth rank	1.36	2.66	*P* < 0.00001
Average NDCG	0.841	0.801	*P* < 0.0001

Chi-squared test was used to calculate significance of the proportion of ground truth documents in top 3, and Mann–Whitney *U* test was used for ground truth rank and NDCG. All metrics show that the algorithm performed significantly better on simple indications than on complex ones. Normalized discounted cumulative gain (NDCG), Appropriateness criteria (AC)

Next, we evaluated our algorithm on Testing Dataset 2, true radiology report clinical indications from our institution. Our algorithm ranked the appropriate document as within the top 3 search results for 86% of indications for indications with one appropriate document (Table 3). For indications with multiple appropriate documents, 78% had all documents ranked within the top 5. There were 23 indications with no matching appropriate AC document(s), with a roughly even split between the indication being too vague (e.g. “Trauma”) and no ACR document existing for the indication (e.g. multiple sclerosis, surveillance of various malignancies). Examples of algorithm performance are provided in Additional file 1.

**Table 3:** Institutional radiology report clinical indications dataset results

Classifications and metrics	Number of documents (proportion)
**Single matching doc** (n=59)	
Correct doc ranked top 3	51 (0.864)
Correct doc ranked top 10	57 (0.966)
**Multiple matching docs** (n=18)	
All correct docs ranked top 5	14 (0.777)
All correct docs ranked top 10	18 (1.0)
**No matching doc** (n=23)	
Inadequate indication	11 (0.478)
No AC doc for indication	12 (0.522)

Normalized discounted cumulative gain (NDCG), Appropriateness criteria (AC)

### Comparison to google search engine on AC documents

**Fig. 5:**
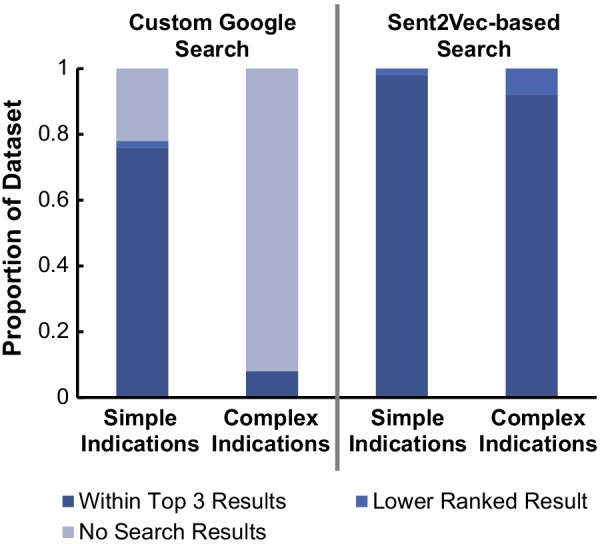
Comparison to a custom google search. This figure shows the relative accuracies of our proposed Sent2Vec-based algorithm and a custom Google search engine on a subset of the simulated indications dataset (n = 100). A lower ranked search result was defined as the ground truth AC document being ranked 4th highest or worse. Document retrieval performance between search engines is statistically significant for both simple and complex indications (*p* < 0.0001 for both indication types, Friedman Rank Test)

To compare our Sent2Vec algorithm’s performance to a more traditional free-text algorithm, we created a custom Google search engine constrained to searching on the 205 AC documents. As shown in Fig. 5, Google search yielded results for 5% of complex simulated indications that resemble radiology report clinical indications, while our algorithm yielded results for 100% of queries, with top three accuracy for 90%. Similarly, for the simple simulated indications, our algorithm provided search results for all queries with top three accuracy of 98%, while Google search provided results for 78% of queries.

### Error analysis

Table 4 illustrates indications with the lowest ranking of correct AC document(s) in our two datasets. Each of these errors showcases a different imperfection with the algorithm, AC corpus, or the input query. Error 1 in the simulated indications was largely due to poor semantic similarity of “big toe pain” to a key clinical diagnosis, “gout”, which was well represented in the ground truth document. Replacing “big toe pain” with “podagra” results in a top 3 ground truth ranking.

**Table 4:** Error analysis

Dataset and indications	Ground truth document	Ground truth rank	Main cause of error
*Simulated indications dataset*			
1. “64yo woman with history of obesity and alcohol use disorder presents with chronic onset of progressive big toe pain and swelling.”	Chronic extremity joint pain, suspected inflammatory arthritis	10	Semantic matching of “big toe pain”
2. “60yo female with history of hypertension presents with right groin pain, fatigue, and weight loss for past 3 months. Concerning for sarcoma”	Soft-tissue masses	54	No clinical context in ground truth document
3. “21yo G2P0A1 with history of recent termination procedure presenting with vaginal bleeding and vomiting for a week”	Gestational trophoblastic disease	15	Vague indication
*Institutional radiology report indications dataset*			
1. “Aortic dissection suspected. Cancer metastatic pt with pancreatic cancer stage IV currently on treatment and needs restaging scan”	Acute chest pain–suspected aortic dissection	Not top 10	Overly specific indication with distracting medical history

In Error 2, the most important word was “sarcoma”, but it was accompanied by a significant amount of distracting clinical history and symptoms that were not mentioned in the ground truth AC document. Therefore, the algorithm calculated better semantic similarity of this indication with other malignancy documents that do detail clinical symptoms. The direct word “sarcoma” does appear in document text, but thesent2vec attempts to capture a semantic approach that dilutes the direct word mentions.

Error 3 had a vague indication, which caused more general Ob/Gyn documents (e.g. “Abnormal Vaginal Bleeding”) to become prioritized. In contrast, error 1 from the radiology report dataset was from an indication too specific in detailing two different contexts, one of aortic dissection and one of pancreatic cancer. The algorithm was not able to recognize that the aortic dissection would be the more clinically urgent part of the indication.

## Discussion

To facilitate the use of the best-practice driven AC, we have developed a sent2vec based search engine leveraging PubMed and MIMIC-III datasets that incorporates semantic similarity and TF-IDF features. Our evaluations on comprehensive simulated and radiology report datasets showed high document retrieval accuracy and relevance. Specifically, 98% of simple and 85% of complex simulated indications had ground truths ranked in the top 3, and 86% of clinical indications with one appropriate document had the same ranking.

Unlike in other machine learning tasks, evaluating a query algorithm on the AC corpus is inherently challenging because a clinical indication does not always have one most relevant AC document, as many documents overlap heavily and can only be differentiated with additional information that is often unavailable (e.g. “Abnormal Vaginal Bleeding” and “First Trimester Vaginal Bleeding”). However, a subgroup of document(s) is obviously more appropriate than others. We addressed this issue of multiple documents being equally appropriate by considering a query search correct if the ground truth document is in the top three results. Furthermore, we divided the clinical report dataset into indications with multiple and single appropriate documents at the discretion of the evaluating clinical radiologist, and separately evaluated each subdivision.

Our algorithm performed well on institutional clinical indications and simulated complex indications, both of which resemble true clinical indications from radiology reports. This high performance implies that true radiology report clinical indications from the EHR can be directly input to the algorithm to provide appropriate imaging guidelines. In our review, we found that no existing AC search methods are successful with similar input. Our algorithm can be integrated into EHR systems to automatically provide clinicians with imaging recommendations when they are inputting the mandatory clinical indications field in imaging orders.

Higher retrieval rate compared to a custom Google search engine suggests that our algorithm has been finetuned for interpreting radiology report clinical indications, likely secondary to being trained on a biomedical corpus. In contrast, Google search is tuned to search shorter queries that span multiple knowledge fields. Therefore, the difference in performance, especially on EHR-style complex simulated indications, is likely attributable to our algorithm’s specialized tuning.

Among many potentially applicable semantic similarity NLP tools, we chose sent2vec because it was reported to perform better on clinical datasets than word2vec, doc2vec, levenshtein distance, and universal sentence encoding [18]. Although it has outperformed many other NLP models, the Bidirectional Encoder Representations from Transformers (BERT) model’s training methodology of next sentence prediction [22] was felt unlikely to perform well on equating the contexts of long documents and short queries. In contrast, sent2vec contextually represents concepts and has been successfully applied to scientific sentiment analysis [12, 23]. Though its large vector size of 700 helps to combat dilution of information during document averaging, we also incorporated a header vector based on document title and variants to ensure important clinical concepts are not lost. Finally, we improved differential document retrieval by adding TF-IDF features that highlight each document’s key points.

Combining TF-IDF and word embeddings is considered superior to state of the art methods for text classification [24, 25] and has shown success in information retrieval [13]. However, disadvantages of previous implementations that used cosine distance between raw TF-IDF vectors were that semantically equal words/phrases were treated as separate features and that TF-IDF models had limited features, especially in smaller datasets like the AC corpus (TF-IDF: ~ 50,000 vs. sent2vec: ~ 5 million). We accounted for these issues by embedding each document’s top TF-IDF features using our sent2vec model, giving a broad semantic vocabulary to the corpus information.

Our algorithm had several limitations. First, the algorithm's matching performance may be decreased for concepts with very low frequencies in the training MIMIC-III and PubMed datasets. Second, the algorithm is inherently limited by the comprehensiveness of the model’s vocabulary list, albeit quite extensive currently at ~ 3 million words. Third, the matching ability is also limited by the breadth of the AC corpus. Some clinical indications (12% of radiology report dataset) are not covered by any AC topic document, and some AC documents lack clinical context discussions that could semantically match to indication queries. Fourth, the algorithm is optimized to match the semantic relevance of the free-text clinical indication sentence rather than to exactly match each word with AC content. This was done to suppress the effect of distracting language within query indications, which was typical in our institutions’ imaging orders. However, if the query indication does not have as much distracting language, the exact TF-IDF matching of the query terms could be emphasized over semantic matching with sent2vec by tuning hyperparameters.

Future work would necessitate recruiting clinicians to use a system based on our algorithm when ordering scans so that we can further refine the algorithm. Furthermore, integrating demographic information and applying NLP question generation techniques to pose questions to users could allow for query refinement and matching to specific variants.

## Conclusions

In summary, a natural language processing algorithm was developed to allow clinicians access to automatically searched radiology recommendations from the American College of Radiology’s Appropriateness Criteria when they order imaging. This algorithm shows promise for further testing and integration into clinical workflow as an automated decision support tool to assist clinicians with choosing appropriate radiological studies. Beyond the AC, our fully opensource algorithm can be readily developed into a high-performing semantic search engine on other biomedical corpuses.

## Supplementary Information

**Additional file 1. Supplemental Table 1.** Example indications and corresponding algorithm performance.

## Data Availability

The generated dataset used for testing in the current study is available on GitHub at https://bit.ly/3giZwSa. The retrospectively collected radiology reports dataset is not publicly available due to HIPPA regulations.

## Abbreviations

ACR: American College of Radiology
AC: Appropriateness Criteria
NLP: Natural language processing
TF-IDF: Term frequency inverse document frequency
NDCG: Normalized discounted cumulative gain
