# Supplementary material for: Clinical language search algorithm from free-text: facilitating appropriate imaging
Source: BMC Med Imaging. 2022 Feb 4;22:18. doi: 10.1186/s12880-022-00740-6 (PMC8815252; doi:10.1186/s12880-022-00740-6)
Supplement: Supplementary file 1 — Additional file 1. Supplemental Table 1. Example indications and corresponding algorithm performance. [file 12880_2022_740_MOESM1_ESM.docx]

# Supplemental Data

Supplemental Table 1. Example indications and corresponding algorithm performance

| Dataset, Indication Type, and Indication | Ground Truth Document(s) | Ground Truth Rank |
| --- | --- | --- |
| **Simulated Dataset** |  |  |
| Simple Indications |  |  |
| “sinusitis and epistaxis, sinonasal tumor suspected.” | Sinonasal Disease | 1 |
| “pain and swelling after total hip replacement” | Imaging after Total Hip Arthroplasty | 1 |
| “TAVR planning” | Imaging for Transcatheter Aortic Valve Replacement | 1 |
| Complex Indications |  |  |
| “70yo with history of hypertension and alcohol use disorder presents with acute episodes of melena. Colonoscopy positive for variceal bleeding. MRI shows thrombus in spleen.” | Radiologic Management of Gastric Varices | 1 |
| “3yo presenting with altered mental status, complex skull fracture, and skin bruises.” | Suspected Physical Abuse-Child | 1 |
| “54yo with history of medically managed HIV presents with acute onset fever, dyspnea, and productive cough” | Acute Respiratory Illness in Immunocompromised Patients | 4 |
| **Institutional Radiology Report Dataset** |  |  |
| Single Appropriate AC Document |  |  |
| “Left thalamic vascular lesion, trigeminal neuralgia, right upper extremity, history of breast cancer.” | Cranial Neuropathy | 2 |
| “Chronic right periorbital edema, facial pain, prior history of periorbital abscess with drainage and diagnostic testing negative for granulomatous disease.” | Orbits, Vision and Vision Loss | 1 |
| Multiple Appropriate AC Documents |  |  |
| “please eval for torsion and possible appy, has known large ovarian mass with RLQ to groin pain” | Right Lower Quadrant Pain-Suspected Appendicitis;  Acute pelvic pain in the reproductive age group | 1, 4 |
| “20yo with R femur lesion c/f osteomyelitis vs malignancy, had non-con MR, per Orthopedics needs post-contrast scan to determine whether biopsy is indicated” | Suspected Osteomyelitis, Septic Arthritis, or Soft Tissue Infection (Excluding Spine and Diabetic Foot);  Primary bone tumors | 1, 2 |
